# A pilot-scale forward osmosis membrane system for concentrating low-strength municipal wastewater: performance and implications

**DOI:** 10.1038/srep21653

**Published:** 2016-02-22

**Authors:** Zhiwei Wang, Junjian Zheng, Jixu Tang, Xinhua Wang, Zhichao Wu

**Affiliations:** 1 State Key Laboratory of Pollution Control and Resource Reuse, School of Environmental Science and Engineering, Tongji University, Shanghai, 200092, P.R. China; 2 School of Environmental and Civil Engineering, Jiangnan University, Wuxi 214122, P.R. China

## Abstract

Recovery of nutrients and energy from municipal wastewater has attracted much attention in recent years; however, its efficiency is significantly limited by the low-strength properties of municipal wastewater. Herein, we report a pilot-scale forward osmosis (FO) system using a spiral-wound membrane module to concentrate real municipal wastewater. Under active layer facing feed solution mode, the critical concentration factor (CCF) of this FO system was determined to be 8 with 0.5 M NaCl as draw solution. During long-term operation at a concentration factor of 5, (99.8 ± 0.6)% of chemical oxygen demand and (99.7 ± 0.5)% of total phosphorus rejection rates could be achieved at a flux of 6 L/(m^2^ h) on average. In comparison, only (48.1 ± 10.5)% and (67.8 ± 7.3)% rejection of ammonium and total nitrogen were observed. Cake enhanced concentration polarization is a major contributor to the decrease of water fluxes. The fouling also led to the occurrence of a cake reduced concentration polarization effect, improving ammonium rejection rate with the increase of operation time in each cycle. This work demonstrates the applicability of using FO process for wastewater concentrating and also limitations in ammonium recovery that need further improvement in future.

Currently, wastewater is increasingly considered as a source of water, nutrients and energy rather than a waste^1^,^2^. For nutrients and energy recovery from domestic/municipal wastewater, a major barrier is the low-strength nature of wastewater which significantly impacts its recovery efficiency and cost-effectiveness. To provide a concentrate with high concentrations of chemical oxygen demand (COD) and nutrients (nitrogen and phosphorus) that meet the economic benefits holds the key to the down-stream energy capture (e.g., anaerobic treatment and microbial fuel cells) and nutrient recovery units^3^.

Membrane separation is a promising technology for the concentration purpose. Aerobic membrane bioreactors (MBRs) with short hydraulic retention time (HRT) and short sludge retention time (SRT) have been used for concentrating sewage and grey water through bioflocculation mechanisms^4^,^5^. The major drawback of this scenario is severe membrane fouling and *in-situ* COD biodegradation during the concentrating process (resulting in only about 35% COD recovered)^4^. Dynamic membrane separation developed by Ma *et al.*^6^ demonstrated an 81.6% of COD recovery rate under a high membrane flux of 60 L/(m^2^ h). Direct sewage up-concentration by microfiltration (MF) membranes has been also reported^3^, and efficient concentration was achieved for COD, but not for nitrogen and phosphorus. Nanofiltration (NF) and reverse osmosis (RO) can be also used for concentrating municipal wastewater^7^,^8^; however, NF and RO membranes are sensitive to fouling by dissolved and undissolved molecules, particulate matter, salt precipitates and microorganisms^9^,^10^,^11^. For this reason, NF and RO systems for wastewater treatment require pretreatment to reduce membrane fouling, e.g., MF and ultrafiltration (UF) as pretreatment steps^12^.

Forward osmosis (FO) is a membrane separation process with a semi-permeable membrane placed between a feed solution (FS) of a low osmotic pressure and a draw solution (DS) of high osmotic pressure, and is driven by the osmotic pressure difference across the membrane^13^. The FO process presents lower fouling propensity compared to traditional pressure-driven membrane processes such as NF and RO, and thus has attracted much attention in recent years^14^,^15^,^16^,^17^. Use of FO processes for low-strength domestic/municipal wastewater treatment is steadily increasing, e.g., synthetic domestic wastewater^16^, and wastewater effluent from municipal sources^18^,^19^ and municipal wastewater^20^,^21^. The above-mentioned studies lay the groundwork for understanding the behaviours of FO systems for concentrating wastewater; however, it is still insufficient to establish a general rule for these systems since most of the studies use lab-scale FO systems under batch-filtration mode and the experimental duration lasts for several hours to several days^18^,^19^,^20^,^21^. A long-term investigation of FO systems under continuous flow operation for concentrating low-strength domestic/municipal wastewater is in great need of in order to push forward the applications of this technology to real wastewater treatment.

In the present work, we established a pilot-scale FO membrane system using a spiral wound FO membrane module with an effective area of 0.3 m^2^ for concentrating real municipal wastewater. The critical concentration factor (CCF) was first determined, and long-term performance of this pilot-scale FO system at a concentration factor (CF) of 5 was then investigated. The contribution of external concentration polarization (ECP), cake enhanced concentration polarization (CECP) and solute back-diffusion to the decrease in flux performance was analyzed, and the role of cake reduced concentration polarization (CRCP) in ammonium rejection was also discussed. The obtained results are expected to provide a sound understanding on FO systems for concentrating low-strength wastewater.

## Results and Discussion

### Membrane permeability using DI water as feed solution

**Figure 1:**
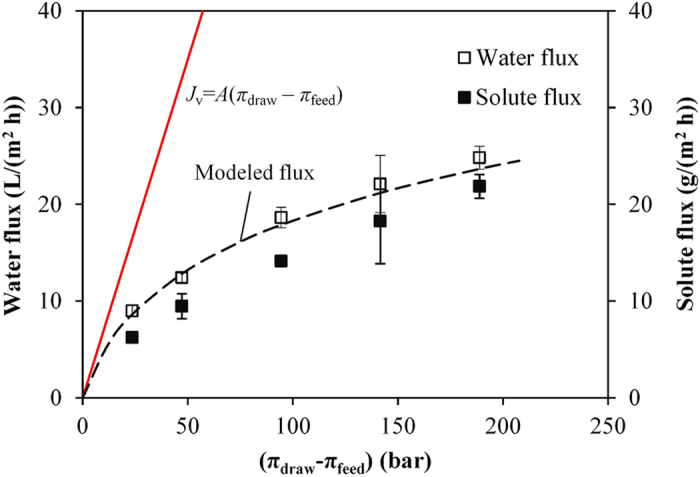
Water and solute fluxes as a function of osmotic pressure using DI water as feed solution. The red solid line is the modeled flux using *J*_v_ = *A*(π_draw_ − π_feed_), and the black dashed line indicates the modeled flux using Eq. (1). The square symbols represent the measured data. *Error bars* represent standard deviations; where absent, bars fall within symbols.

The intrinsic *A* and *B* parameters of this CTA membrane used in this study were determined to be 0.70 ± 0.07 L/(m^2^ h bar) and 0.53 ± 0.03 L/(m^2^ h), respectively, which are similar to previous publications^22^,^23^. The water and solute fluxes (using DI water as feed solution) as a function of osmotic pressures are shown in Fig. 1. The measured water and solute fluxes increase with the increase of osmotic pressure; however, the water fluxes of CTA membranes deviate from the theoretical flux using the linear curve (*J*_v_ = *A*(π_draw_ − π_feed_)) based on the classical solution-diffusion theory^13^ but can be well modeled by Eq. (1), indicating that ICP^24^ can significantly impact the water fluxes. The mass transfer coefficient of this CTA membrane, *K*_m_, which is related to the ICP phenomenon within the porous support layer, was modeled to be (4.07 ± 0.26) × 10^−6^ m/s. The *K*_m_ value obtained in this study is in good agreement with the value reported by Tang *et al.*^25^ for the same kind of membrane (4.2 × 10^−6^ m/s for the AL-FS configuration) with a CFV of 23.2 cm/s. The structure parameter, *S*_me_, was calculated to be (2.96 ± 0.26) × 10^−4^ m using a *D*_draw_ value of 1.2 × 10^−9^ m^2^/s for 0.5 M NaCl at 20 °C^23^. The *S*_me_ value (296 μm) was much larger than the support layer thickness (39 ~ 51 μm)^26^,^27^,^28^, which is attributed to the support layer’s tortuosity and porosity. The *K*_m_ value can be used in the fouling-incorporated water flux model (Eq. (3)) for evaluating the performance of this pilot-scale FO membrane system for concentrating real wastewater.

### Critical concentration factor (CCF) for concentrating wastewater

**Figure 2:**
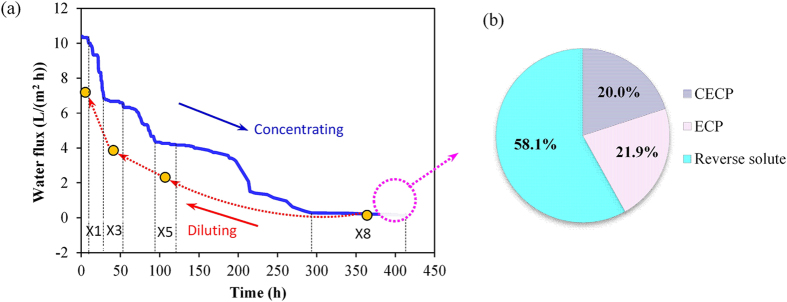
(**a**) Changes of water fluxes during concentrating wastewater for determining CCF. The solid blue line represents the variations of water fluxes for continuous concentration of municipal wastewater, while the yellow circles indicate the water fluxes at respective concentrating factors through step-wisely diluting the concentrated wastewater by DI water. (**b**) The contribution of CECP, external concentration polarization (ECP) and reverse solute to water flux decrease at CCF. X1, X3, X5 and X8 indicate that the concentration factors (CF) are 1 time, 3 times, 5 times and 8 times that of influent sewage.

Variations of water fluxes during the determination of CCF are shown in Fig. 2, and the corresponding solute fluxes are illustrated in Fig. S1 in the Supplementary Information (SI). The water fluxes are gradually decreased due to membrane fouling and solute back-diffusion^25^, and the solute fluxes show similar changing pattern (see supplementary Fig. S1). The CCF of this pilot-scale FO system for concentrating municipal wastewater was determined to be 8, indicating that this FO system should be operated with CF less than 8, i.e., a sub-critical CF, for achieving a cost-effective performance. Step-wise diluting of the concentrated wastewater did not restore the water fluxes back to those in the concentrating process, indicating that membrane fouling together with solute back-diffusion made the flux behaviours irreversible. However, the solute fluxes during step-wise diluting were very close to those in the concentrating process (see Fig. S1). In order to further examine the impacts of membrane fouling on water permeability and to explain the differences between water and solute flux changing behaviours, the fouling-incorporated water flux model (Eq. (3)) was used to evaluate the obtained data. The osmotic pressures of feed solutions at different CF during step-wise diluting process were measured, which are summarized in supplementary Table S1. Using this model and measured data, the parameters related to water and solute fluxes at the CCF could be calculated, and the results are listed in Table 1.

From Table 1, it can be observed that *A* is decreased to 0.582 L/(m^2^ h bar) from its original value 0.70 L/(m^2^ h bar), indicating that membrane fouling resulted in an increased hydraulic resistance of the fouled membrane and thus a decreased water permeability^25^. However, it is very interesting to observe that the *B* value present no obvious change compared to the virgin membrane. This leads to the increase of the overall *B*/*A* ratio, indicating that a serious fouling occurs as reported by Lay *et al.*^29^. The *A*_la_ value is much less than the *B*_la_ value (Table 1), suggesting that the fouling layer formed on AL of FO membranes has a poor selectivity and thus negligible impacts on reverse salt rejection compared to its influence on water permeability during the CCF test. It can well explain that many authors observed a less significant decrease in solute fluxes compared to a dramatic decrease of water fluxes when fouling happened in FO systems^26^,^30^.

Figure 2(b) shows the contribution of various factors to the decrease of membrane permeability at CCF (detailed calculation shown in supplementary material). Since the draw solution concentration was maintained constant, ICP was thus thought to remain approximately unchanged. Therefore, only the phenomenon occurring on the feed solution side was taken into consideration, namely CECP, external concentration polarization (ECP) and solute back-diffusion. It is clear that the solute back-diffusion dominated the decrease of water flux during the concentrating process, while the CECP and ECP contributions were similar. The accumulated salinity can reduce the effective osmotic pressure difference available for driving the water flux through FO membrane in the whole concentrating process, which is also regarded as a major reason causing the deterioration of FO performance in forward osmosis membrane bioreactors^31^,^32^,^33^.

### Long-term performance of FO membrane for concentrating wastewater

**Figure 3:**
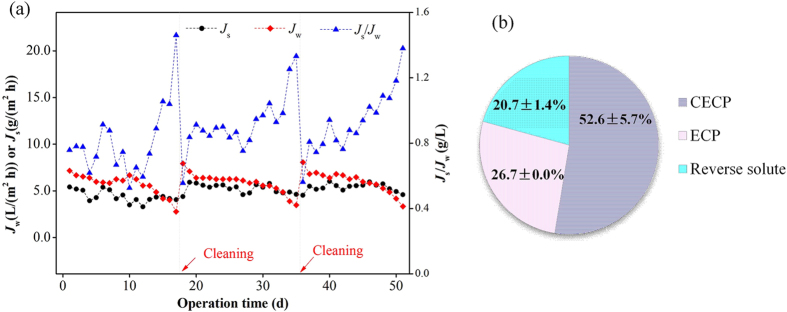
(**a**) Variations of water and solute fluxes, and solute to water flux ratio during the long-term operation of this pilot-scale FO system for concentrating municipal wastewater at CF 5; (**b**) The contribution of CECP, ECP and reverse solute to water flux decrease at the points of membrane cleaning. Cleaning procedure is described in Materials and Methods.

Based on CCF results, the CF of 5, i.e., a sub-critical CF, was chosen for the FO pilot-scale system, and long-term performance of the FO membrane was examined during concentrating municipal wastewater. In total, three cycles lasting for 51 d were performed, and changes of water and solute fluxes during the operation are shown in Fig. 3(a). It can be observed that the water fluxes in each cycle showed a three-step changing pattern, i.e., a rapid decrease in the initial filtration stage (from 7.7 L/(m^2^ h) to 6.5 L/(m^2^ h) on average), a slow decrease stage (about 6 L/(m^2^ h) on average), and followed by a rapid decrease again at the end of a cycle. The rapid decrease in water fluxes in the initial stage might be due to the rapid formation of external concentration polarization on the feed solution side (resulting rapid reversible fouling of FO membrane) and the internal concentration polarization on the draw solution side^34^. In the second stage, membrane fouling layer was gradually formed, resulting in the occurrence of cake enhanced concentration polarization (or termed cake enhanced osmotic pressure). Afterward, fouling (cake) layer reached a critical condition after a long-term accumulation (e.g., the dramatic increase of thickness, compressibility and CECP effects), causing a rapid decrease in water fluxes again at the end of each cycle. However, the solute fluxes were kept relatively stable during the filtration process and tended to decrease slightly at the end of one filtration cycle. This suggests that the impacts of membrane fouling on solute fluxes are less significant compared to water fluxes, which is consistent with the results of CCF test. That is why the ratio of solute fluxes to water fluxes (*J*_s_/*J*_w_) increases dramatically at the end of each filtration cycle. Larger ratios of *J*_s_/*J*_w_ reflect a decrease in the selectivity of the overall membrane (including fouling layer) and lower efficiency of the process^35^. During the operation in each cycle, the salt concentration in terms of total dissolved solids (TDS) in the feed solution ranged from about 6.1 g/L in the initial stage to around 4.2 g/L in the later stage, suggesting that the salt concentration was not accumulated in the concentrating process due to the periodical discharge of concentrated wastewater from the feed solution tank. The decrease in the salt concentration in the later stage of each cycle was mainly attributed to the rapid decrease of water fluxes (Fig. 3) and CF.

The specific contributions of CECP, ECP and reverse solute diffusion to the decrease of membrane permeability were determined, and the results are shown in Fig. 3(b). It is evident that during long-term operation the CECP is the major factor impacting the water fluxes, followed by ECP and solute back-diffusion. It is much different from CCF test as shown in Fig. 2(b). This is because the accumulation of solute in the FO system during the long-term operation was significantly alleviated by periodically discharging the concentrated wastewater. The formed fouling layer in FO systems during long-term operation is reported to be irreversible and chemical cleaning is needed for recovering the permeability^26^,^36^,^37^.

**Figure 4:**
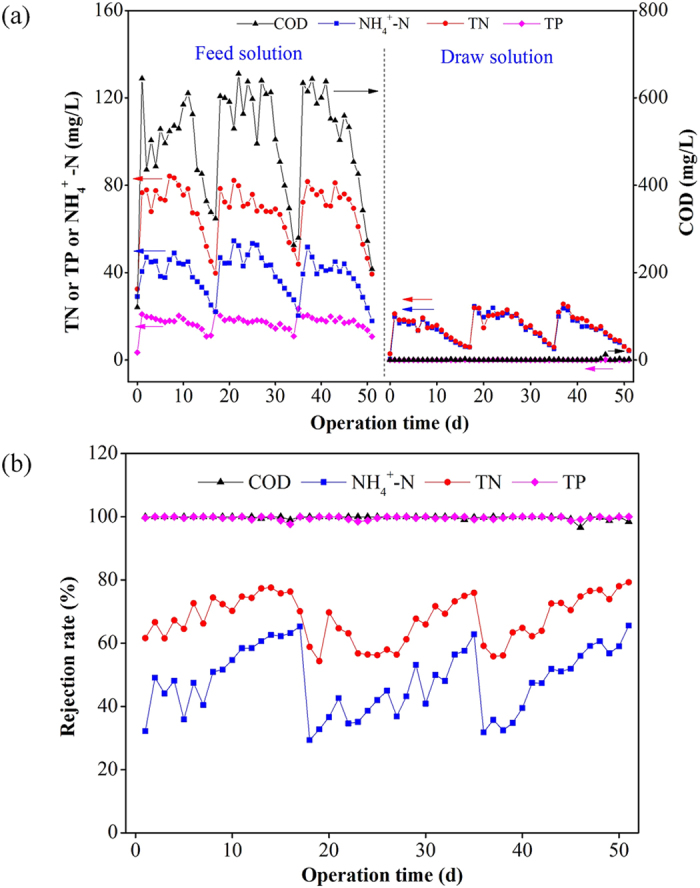
(**a**) Pollutant concentrations in feed solution and draw solution; (**b**) Rejection rates of pollutants in the FO system during long-term operation.

The rejection of pollutants existing in wastewater is an important factor reflecting the concentrating efficiency. Figure 4 illustrates the variations of pollutant concentrations in feed and draw solutions and also the changes of rejection rate during the long-operation. From Fig. 4, it is clear that the pilot-scale FO system could achieve (99.8 ± 0.6)% of COD and (99.7 ± 0.5)% of TP rejection rates. However, only (48.1 ± 10.5)% and (67.8 ± 7.3)% rejection of NH_4_^+^-N and TN were observed during this concentrating process, respectively. The low rejection rate of ammonium is attributed to bidirectional diffusion of ammonium of feed solution and sodium cations of draw solution in forward osmosis process^38^. Since TN in the feed solution also contained part of organic nitrogen except ammonium, the rejection rate of TN was therefore higher compared to NH_4_^+^-N due to the sound rejection of organic matters by the FO membrane.

**Figure 5:**
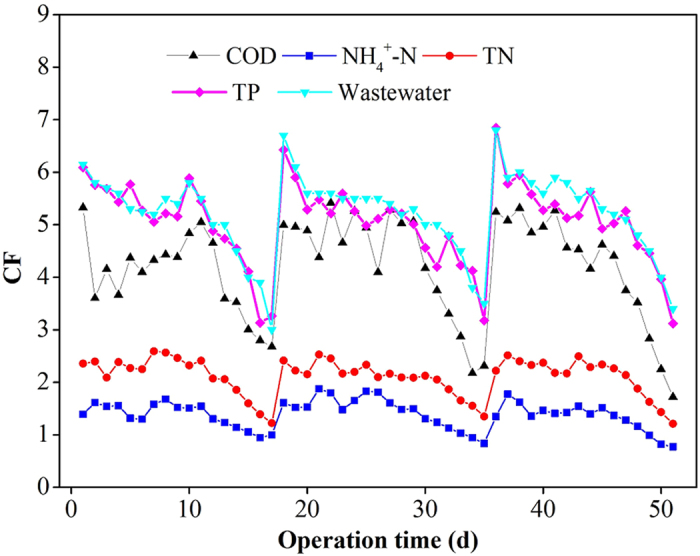
Concentration factors (CF) of wastewater and pollutants in the pilot-scale FO system during long-term operation. The initial CF higher than 5 for each cycle is due to the complexity of system control such as the influent water level and the variation of membrane permeability after cleaning.

As discussed earlier, the FO membrane achieved different rejection rates for various pollutants although a pre-determined CF of 5 was used. Therefore, the CF values for wastewater and various pollutants might be different during the long-term operation, which were further calculated and are plotted in Fig. 5. The CF values of COD, TP, TN and NH_4_^+^-N are all less than the CF of wastewater. This is because that the FO membrane presented different rejection behaviours for various pollutants. For a long-term operation, the concentrating efficiencies for ammonium and total nitrogen in the FO system were lower compared to COD and TP. Development of modified FO membranes to suppress the diffusion of monovalent ions (ammonium) across FO membranes should be carried out for achieving a reasonable rejection^38^. Another limitation for concentrating wastewater is related to the biodegradation of organic matters although the degradation rate is much slower compared to other bioflocculation method^4^. In order to further understand the concentrating efficiency, mass balance analysis was carried out, which is shown in Fig. S2. Take COD as example, about 19.2% of COD was degraded or attached to membrane surfaces to form a fouling layer for each operation cycle. Nevertheless, in our study, the final COD concentration could reach 2335 ± 146 mg/L by mixing the concentrated wastewater (at a CF of 5) and the recovered particulate/colloidal matters in the pretreatment unit. According to the theoretical energy potential value of 3.86 kW h/kg COD and current energy conversion efficiency of 28% in literature through methane recovery and combustion^1^, the obtained electricity potential for the concentrated wastewater is about 2.52 kW h/m^3^-wastewater. Currently, a typical anaerobic treatment (with 80% removal rate) and a down-stream aerobic treatment of this concentrated wastewater for meeting the wastewater discharge standard consumes about 0.4 kW h/m^3^ and 0.6 kW h/m^3^ using state-of-the-art technologies, respectively^1^, with a total energy consumption of about 1.0 kW h/m^3^. The energy-neutral point using this treatment scenario is achieved at the concentrated COD concentration of about 925 mg/L. This indicates that the COD level of this study using FO concentration (2335 mg/L on average) could sufficiently meet the economic benefits.

**Figure 6:**
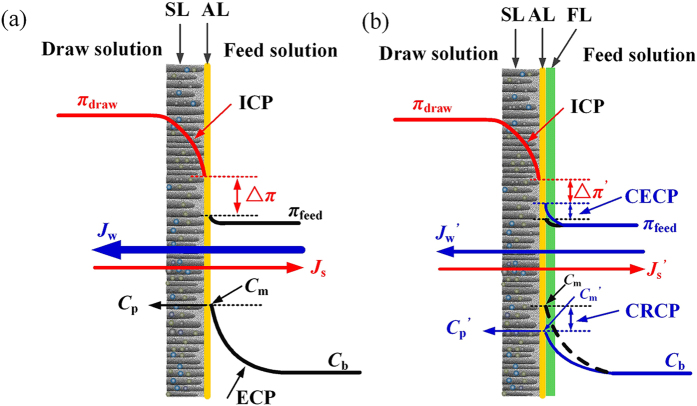
Illustration of concentration polarization and fouling for FO membrane and their impacts on water/solute fluxes and ammonium rejection in this study. (**a**) FO membrane in the initial filtration; (**b**) FO membrane with fouling layer formed. Note: SL, support layer; AL, active layer; FL, fouling layer; ICP, internal concentration polarization; ECP, external concentration polarization; CECP, cake enhanced concentration polarization; CRCP, cake reduced concentration polarization.

It is interesting to observe that the rejection rate of ammonium is increased as a function of operation time. In order to explain this phenomenon, the concentration polarization model, as shown in Eq. (7), was used to process the data, and the results are summarized in Table 2. The mass transfer coefficient was decreased, and the ammonium concentration at the membrane interface (*C*_m_) was also lowered at the ending stage compared to those at the initial stage (also illustrated in Fig. 6). This is related to the formation of fouling layer on FO membrane surface, which more significantly hindered convection mechanism than diffusion mechanism. Thus, the concentration on the membrane surface was lower (see Fig. 6(b)) than what was expected for a normal concentration polarization attributed to convection and diffusion (Fig. 6(a)). This phenomenon can be termed cake reduced concentration polarization (CRCP), which has been observed in seawater reverse osmosis system (SWRO) processes^39^. The lower ammonium concentration as shown in Table 2 on membrane surface (*C*_m_) for the fouled FO membrane compared to the clean membrane, confirming the occurrence of CRCP in our study. The low *C*_m_ consequently resulted in the improvement of rejection rate compared to normal concentration polarization attributed to convection and diffusion. Similarly, CRCP may also improve flux performance. However, the positive impact of CRCP on water fluxes is much less significant compared to the negative impact of CECP^29^. Therefore, CRCP is negligible when flux behaviours are evaluated. In summary, as illustrated in Fig. 6, the fouling layer formed on AL of FO membrane resulted in a decrease in osmotic pressure difference and consequently a reduction of water permeability. In addition, the fouling layer, due to its poor selectivity, had less significant impacts on solute fluxes compared to water fluxes, leading to an increase of *J*_s_/*J*_w_ during the long-term operation. However, for the rejection of ammonium, the fouling layer induced a CRCP phenomenon, improving the rejection performance with the increase of operation time in each cycle.

### Implications of this work

Although the concept of using forward osmosis membrane to concentrate municipal wastewater has been proposed for energy and nutrients recovery in recent years^18^,^21^,^40^, its applicability is not systematically evaluated at pilot-scale or full-scale operation yet. This work provides the evidence of using FO membrane for concentrating dilute wastewater on a pilot-scale for the first time. It demonstrates that a critical concentration factor exists and a sub-critical concentration factor should be used in this system for achieving a cost-effective treatment. The long-term pilot-scale test also achieved a higher concentration factor compared to bench-scale experiments (usually with CF 2~3) reported by others^18^,^21^, demonstrating its promising prospect for dilute wastewater treatment and resource recovery.

This pilot-scale test also confirms that the currently available FO membrane can obtain highly efficient rejection of organic matter and phosphorus but relatively low separation of ammonium. In order to further enhance the recovery efficiency of ammonium, high-performance FO membranes^38^,^41^,^42^ with high water permeability and low solute permeability should be developed to suppress the bidirectional diffusion of ammonium and sodium cations during the FO process. This existing challenge calls for intensive interdisciplinary collaborations between material scientists and environmental engineers. Modification of surface charge and functional groups for FO membranes to improve their selectivity for cations should be explored in future.

## Materials and Methods

### Experimental set-up and FO operation

**Figure 7:**
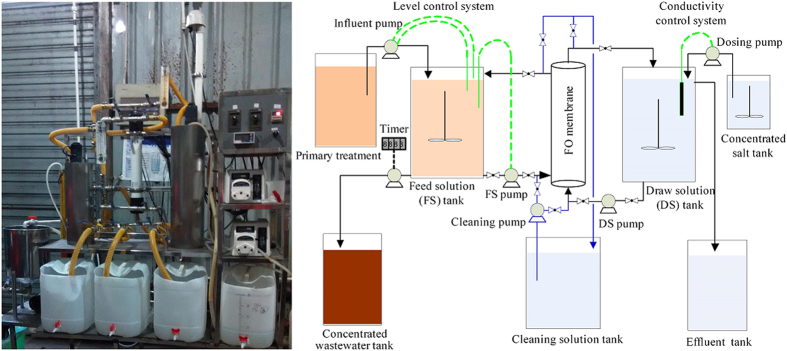
Photograph (left) and schematic representation (right) of this FO system.

The pilot-scale FO system, as shown in Fig. 7, was located in Quyang Municipal Wastewater Treatment Plant (WWTP), Shanghai, China and used for concentrating real municipal wastewater. It consisted of a primary treatment unit, a feed solution (FS) tank, a spiral-wound FO membrane module, a draw solution (DS) tank, a cleaning solution tank, a concentrated wastewater tank, a concentrated salt tank and an effluent tank. The objective of primary treatment employing a dynamic membrane (made of coarse-pore materials) separation unit was to remove part of particulate and colloidal substances existing in real municipal wastewater for alleviating membrane fouling in down-stream FO process, and details of this primary treatment can be found in our previous publication^6^. The separated particulate and colloidal substances in the primary treatment unit can be used to generate biogas and energy^43^. In this work, we expect that the primarily separated substances could be mixed with the concentrated wastewater of the FO system to generate energy and recover nutrients. The effluent of this primary treatment unit was pumped into the FS tank. The characteristics of this FS wastewater, i.e., primarily-treated municipal wastewater, are shown in Table 3. A level sensor was used to control the influent and FS pumps (see Fig. 7) for maintaining a constant water level in the FS tank.

A 0.5 M NaCl solution with osmotic pressure about 23.6 bar was used as the DS. Its concentration in the DS tank was maintained relatively constant by automatically dosing a concentrated NaCl solution (5 M) through a dosing pump which was controlled by a conductivity control system keeping the DS conductivity at the level of 47.3 ~ 47.5 ms/cm. The water flux of this FO membrane (*J*_w_) was determined by quantifying the liquid volume in the effluent tank, where the volume of dosed 5 M NaCl solution was excluded, while the solute flux was calculated based on the changes of total dissolved solids (TDS) on the feed solution side and mass balance analysis. The temperature during the experiment was in the range of 18~22 °C.

During long-term operation, chemical cleaning was carried out for this FO system using 1%Alconox +0.8%EDTA^26^ if water flux was decreased to half of the initial. Each cleaning lasted for 10 min at a cross-flow velocity (CFV) of 20 cm/s. After chemical cleaning, a hydraulic cleaning for 10 min at the same CFV was conducted, and then a new cycle of filtration was restarted.

### Membrane samples and membrane characterization

A spiral-wound membrane module (50.8 cm × φ 8.6 cm) made of cellulose triacetate (CTA) with an effective area 0.3 m^2^ was used in this pilot-scale FO system, which was purchased from Hydration Technologies Innovation (HTI, Albany, USA). This membrane module had a spacer with thickness of 2.5 mm on the FS side and a spacer with thickness of 1.5 mm on the DS side for mitigating concentration polarization.

Flat-sheet CTA FO membranes purchased from HTI were also used for examining their intrinsic permeability. Water permeability (*A*), NaCl permeability (*B*) coefficients, and salt rejection rate of the membranes were determined by RO filtration tests at 11 bar as described by Tiraferri *et al.* and Xie *et al.*^23^,^44^. A lower *B*/*A* ratio might indicate a better filtration performance of an FO membrane. In order to characterize the membrane’s permeability under various DS concentrations, water and solute fluxes were determined in a filtration cell using NaCl solution (from 0.5 M to 4.0 M) as the DS and deionized (DI) water as the FS according to the protocols of a previous publication^26^. The cross-flow velocity (CFV) was maintained at 20 cm/s during the tests. In this study, only AL-FS orientation with the membrane active layer facing the feed solution was investigated since AL-DS with the membrane active layer facing the draw solution always results in severe membrane fouling for wastewater treatment^16^,^26^.

## Modeling FO performance

### Membrane permeability

An analytical model as shown in Eq. (1), taking the effect of internal concentration polarization (ICP) into consideration^24^, was used to evaluate FO performance under the AL-FS orientation.


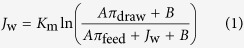


where *J*_w_ is water flux of CTA membrane (L/(m^2^ h)), *A* (L/(m^2^ h bar)) and *B* (L/(m^2^ h)) are intrinsic water permeability and NaCl permeability coefficients, respectively, and π_draw_ and π_feed_ are the osmotic pressure of the draw solution and feed solution (bar), respectively. *K*_m_, the mass transfer coefficient (L/(m^2^ h)), is related to the ICP phenomenon within the porous support layer on the DS side.

*K*_m_ can be worked out using the solute diffusion coefficient *D*_draw_ (m^2^/s) divided by the membrane structure parameter *S*_me_ (m).


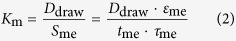


In Eq. (2), *ε*_me_ (−), *t*_me_ (m) and τ_me_ (−) are the porosity, thickness and tortuosity of the membrane support layer, respectively. (−) indicates that it is a dimensionless parameter.

Eq. (1) is valid for well-defined feed (i.e., DI water) under AL-FS orientation for FO membranes^45^, while it may not well simulate the water fluxes in real applications due to the evolution of fouling. Therefore, a fouling-incorporated water flux model for a fouling condition with cake enhanced concentration polarization (CECP) has been developed^46^.


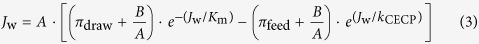


In Eq. (3), *A* (L/(m^2^ h bar)) and *B* (L/(m^2^ h)) are the overall water and salt permeability coefficients, respectively. Their values are dependent on the coefficients of a membrane (subscript ‘me’) and fouling layer (subscript ‘la’), which are shown in Eqs. (4) and (5) 46.


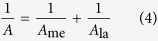



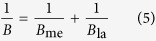


The CECP coefficient, *k*_CECP_ , impacts the permeability of FO membranes during long-term operation. A higher *k*_CECP_ indicates a weaker CECP effect while a lower value shows a more significant effect. Under negligible CECP effects (i.e., *k*_CECP_ = ∞), Eq. (3) can be transformed into Eq. (1).

The relationship of solute flux (*J*_s_) and *J*_w_ can be expressed by the van’t Hoff equation, as shown in Eq. (6) 25.


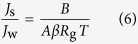


where *β* is the van’t Hoff coefficient (−), *R*_g_ is the universal gas constant (L·bar/(K mol)) and *T* is the absolute temperature (K).

### Concentration polarization impacting ammonium rejection

In the AL-FS orientation for FO system, concentration polarization on the FS side can be characterized by using the boundary layer film theory^47^.


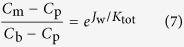


where *C*_b_ (mg/L), *C*_m_ (mg/L) and *C*_p_ (mg/L) are the concentrations of the bulk feed solution, membrane interface and permeate water, respectively. *K*_tot_, the overall mass transfer coefficient (L/(m^2^ h)), which is given by the ratio of solute diffusion coefficient *D*_s_ to the boundary layer thickness *δ*, i.e., *K*_tot_ = *D*_draw_/*δ*.

Since the fouling layer is formed during long-term operation, the mass transfer coefficient, *K*_tot_, includes the mass transfer coefficient of ECP (*K*_ecp_) and the mass transfer coefficient of the fouling layer (*K*_la_), holding the relationship as shown in Eq. (8). For a membrane without fouling layer in initial filtration (*K*_la_ = ∞), *K*_tot_ is equal to *K*_ecp_.


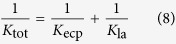


The above-mentioned equations were used in this study to evaluate the rejection behaviours of ammonium during long-term operation.

### Critical concentration factor (CCF) determination

In order to determine the CCF, the pilot-scale FO system was continuously operated under a CFV of 20 cm/s for about 420 h with 0.5 M NaCl solution as draw solution. The draw solution concentration was maintained constant by automatically dosing concentrated salt solution as shown in Fig. 7, while the municipal wastewater was gradually concentrated on the feed side. Due to membrane fouling and solute back-diffusion during this process, the water fluxes were gradually decreased. When the water fluxes were decreased to nearly zero (0.2 L/(m^2^ h) in this study), the concentration factor for the municipal wastewater on the feed side was calculated, which was regarded as CCF in this study. At pre-determined concentration factors (CF), namely 1 time (X1), 3 times (X3), 5 times (X5) and 8 times (X8), the wastewater CF factor was maintained for a period of time by periodically discharging a certain volume of wastewater from the feed solution side in order to examine the permeability of FO membrane at respective CFs.

In order to further examine the contribution of membrane fouling to the decrease of water fluxes, the concentrated wastewater at CCF was gradually diluted by DI water to different CFs, namely 5 times (X5), 3 times (X3), and 1 time (X1). The water and solute fluxes at respective CFs were again determined within 2 h filtration. DI water was also used as feed solution to determine the water and solute fluxes after X1 test was finished. The Eq. (3) was then used to process the obtained data for verifying the impacts of fouling on the permeability. Afterward, the FO membrane was subject to membrane cleaning^26^ as mentioned earlier, and the water and solute fluxes for the cleaned membrane were also measured using DI water as feed solution and 0.5 M NaCl solution as draw solution.

### Long-term operation of this pilot-scale FO system for concentrating wastewater

Based on CCF test, a CF of 5 was chosen for the pilot-scale FO system. Part of the concentrated water was periodically discharged in order to maintain a constant CF. The FO system was operated for 51 d, and if water flux was decreased to half of the initial, chemical cleaning protocol, namely chemical cleaning using 1% Alconox +0.8% EDTA mixture for 10 min followed by hydraulic cleaning for 10 min, was carried out^26^ to recover its permeability. Water and solute fluxes and wastewater characteristics were frequently monitored during this experiment. During the long-term operation, the volume of feed solution and draw solution was maintained at 10 L and 20 L, respectively, using a level sensor system.

Chemical oxygen demand (COD), ammonium (NH_4_^+^-N), total nitrogen (TN) and total phosphorus (TP) in feed and draw solutions were determined according to Standard Methods^48^. The rejection rate (*r*) of these pollutants in the FO system can be calculated by Eq. (9).


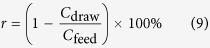


where *C*_draw_ is the pollutant concentration in the draw solution (mg/L) and *C*_feed_ is the pollutant concentration in the feed solution tank (mg/L).

The CF of wastewater in this FO system can be determined by the following equation.


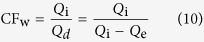


where CF_w_ is the CF of wastewater (−), *Q*_i_ is the influent flow-rate (L/h), *Q*_d_ is the discharging flow-rate of concentrated wastewater (L/h) and *Q*_e_ is the effluent flow-rate of FO membrane (L/h).

Since FO membrane cannot achieve a complete rejection of pollutants existing in wastewater, the CF of pollutants (CF_p_) might be different from the CF of wastewater (CF_w_), and CF_p_ can be worked out by Eq. (11).


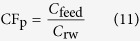


where *C*_rw_ is the pollutant concentration in raw wastewater (mg/L).

## Additional Information

**How to cite this article**: Wang, Z. *et al.* A pilot-scale forward osmosis membrane system for concentrating low-strength municipal wastewater: performance and implications. *Sci. Rep.* **6**, 21653; doi: 10.1038/srep21653 (2016).

## Supplementary Material

Supplementary Information

## Figures and Tables

**Table 1:** Calculated results for the parameters related to water and solute fluxes at the CCF 8.

Parameters	*A* (L/(m^2^ h bar))	*B*(L/(m^2^ h))	*A*_la_ (L/(m^2^ h bar))	*B*_la_ (L/(m^2^ h))	*K*_CECP_ (L/(m^2^ h))
Values	0.582	0.547	3.45	∞^a^	22.1

^a^it means infinity.

**Table 2:** Modeled results for parameters impacting ammonium rejection related to concentration polarization on the feed solution side.

Period	*K*_tot_ (L/(m^2^ h))	*K*_la_ (L/(m^2^ h))	*>C*_m_ (mg/L)
Initial stage	7.30	∞	74.6
Ending stage	4.71	13.27^a^	58.5

^a^*K*_la_ of the FO membrane at the ending stage was calculated by Eq. (8) in which *K*_ecp_ of the ending stage was approximately considered to be equal to its counterpart value of the initial stage, i.e., 7.30 L/(m^2^ h).

**Table 3:** Characteristics of FS (primarily-treated municipal wastewater) used in this FO system (*n* = 10).

FS wastewater	Value
COD (mg/L)	121 ± 33
TN (mg/L)	36.5 ± 5.6
TP (mg/L)	3.4 ± 0.2
NH_4_^+^-N (mg/L)	29.1 ± 5.0
Osmotic pressure (mOsm/kg)	17.5 ± 2.9
TDS (g/L)	1.10 ± 0.16
