# Supplementary material for: A pilot-scale forward osmosis membrane system for concentrating low-strength municipal wastewater: performance and implications
Source: Sci Rep. 2016 Feb 22;6:21653. doi: 10.1038/srep21653 (PMC4761944; doi:10.1038/srep21653)
Supplement: Supplementary Information [file srep21653-s1.doc]

**Supplementary Information**

A pilot-scale forward osmosis membrane system for concentrating low-strength municipal wastewater: performance and implications

Zhiwei Wang^1^,*, Junjian Zheng^1^, Jixu Tang^1^, Xinhua Wang^2^, Zhichao Wu^1^

^1^ State Key Laboratory of Pollution Control and Resource Reuse, School of Environmental Science and Engineering, Tongji University, Shanghai, 200092, P.R. China.

^2^ School of Environmental and Civil Engineering, Jiangnan University, Wuxi 214122, P.R. China.

This Supporting Information contains 2 figures, 1 table and related text for calculating specific contributions of CECP, ECP and solute back-diffusion to membrane permeability decrease at CCF.

*Corresponding Author. Tel./fax: +86-21-65980400; E-mail address: zwwang@tongji.edu.cn (Z. Wang)

X1

X3

X5

X8

Concentrating

Diluting

Fig. S1. Changes of solute fluxes during concentrating wastewater for determining CCF. The solid blue line represents the variations of solute fluxes for continuous concentration of municipal wastewater, while the yellow circles indicate the solute fluxes at respective concentrating factors through manually diluting the concentrated wastewater by DI water.

Fig. S2. Mass balance analysis of pollutants existing in wastewater in this pilot-scale FO system.

Table S1. Osmotic pressures at different CF during step-wise diluting process, and related parameters used for the fouling-incorporated water flux model.

| CF | π_feed_ (bar) | π_draw_ (bar) | *K*_m_ (10^–6^ m/s) | *J*_w_ (L/(m^2^ h)) | Note |
| --- | --- | --- | --- | --- | --- |
| 8 | 22.7 | 23.6 | 4.07 | 0.2 | Fouled membrane |
| 5 | 14.4 | 23.6 | 4.07 | 2.3 | Fouled membrane |
| 3 | 9.2 | 23.6 | 4.07 | 3.9 | Fouled membrane |
| 1 | 2.4 | 23.6 | 4.07 | 7.2 | Fouled membrane |
| 0 (DI) | 0 | 23.6 | 4.07 | 8.2 | Fouled membrane |
| 0 (DI) | 0 | 23.6 | 4.07 | 10.1 | Chemical cleaning |

**SI text: Calculation of specific contributions to membrane permeability decrease at CCF.**

(1) The water flux (*J*_0_) for the clean membrane is about 10.2 L/(m^2^ h) of the spiral-wound membrane at 0.5 M NaCl solution;

(2) The water flux (*J*_e_) for the fouled membrane at CFF is 0.2 L/(m^2^ h).

(3) The water flux (*J*_3_) for the fouled membrane with DI water as feed solution is 8.2 L/(m^2^ h).

Based on the above-mentioned data, the total water flux decrease is 10.0 L/(m^2^ h), i.e., *J*_0_ – *J*_e_. If DI water is used as feed solution for the fouled membrane, the decrease of water flux from 10.2 to 8.2 L/(m^2^ h) is mainly due to the fouling associated with CECP (which may also include pore-blocking of FO membrane). Therefore, water flux decrease due to CECP is equal to 2.0 L/(m^2^ h), i.e., *J*_0_ – *J*_3_.

The raw wastewater has an osmotic pressure about 44.8 kPa (17.5 mOsm/kg). If the solute is not back-diffused, the osmotic pressure at CCF is roughly 8 times that of raw wastewater, i.e., 3.584 bar. Using Eq. (3) as shown in the article, the water flux would be 6.01 L/(m^2^ h) if osmotic pressure of feed solution were 3.584 bar. The flux is labeled as *J*_4_.

Therefore, *J*_4_ – *J*_e_ is equal to the contribution of solute back-diffusion, and *J*_3_ – *J*_4_ should be the contribution of external concentration polarization.
